# Investigating causal pathways in severe falciparum malaria: A pooled retrospective analysis of clinical studies

**DOI:** 10.1371/journal.pmed.1002858

**Published:** 2019-08-23

**Authors:** Stije J. Leopold, James A. Watson, Atthanee Jeeyapant, Julie A. Simpson, Nguyen H. Phu, Tran T. Hien, Nicholas P. J. Day, Arjen M. Dondorp, Nicholas J. White

**Affiliations:** 1 Mahidol Oxford Tropical Medicine Research Unit, Faculty of Tropical Medicine, Mahidol University, Bangkok, Thailand; 2 Centre for Tropical Medicine and Global Health, Nuffield Department of Medicine, University of Oxford, Oxford, United Kingdom; 3 Centre for Epidemiology and Biostatistics, Melbourne School of Population and Global Health, The University of Melbourne, Melbourne, Victoria, Australia; 4 Oxford University Clinical Research Unit, Wellcome Trust Major Overseas Programme, Ho Chi Minh City, Vietnam; Burnet Institute, AUSTRALIA

## Abstract

**Background:**

Severe falciparum malaria is a medical emergency characterised by potentially lethal vital organ dysfunction. Patient fatality rates even with parenteral artesunate treatment remain high. Despite considerable research into adjuvant therapies targeting organ and tissue dysfunction, none have shown efficacy apart from renal replacement therapy. Understanding the causal contributions of clinical and laboratory abnormalities to mortality is essential for the design and evaluation of novel therapeutic interventions.

**Methods and findings:**

We used a structural model causal inference approach to investigate causal relationships between epidemiological, laboratory, and clinical variables in patients with severe falciparum malaria enrolled in clinical trials and their in-hospital mortality. Under this causal model, we analysed records from 9,040 hospitalised children (0–12 years, *n* = 5,635) and adults (*n* = 3,405, 12–87 years) with severe falciparum malaria from 15 countries in Africa and Asia who were studied prospectively over the past 35 years. On admission, patient covariates associated with increased in-hospital mortality were severity of acidosis (odds ratio [OR] 2.10 for a 7-mEq/L increase in base deficit [95% CI 1.93–2.28]), renal impairment (OR 1.71 for a 2-fold increase in blood urea nitrogen [95% CI 1.58, 1.86]), coma (OR 3.59 [95% CI 3.07–4.21]), seizures (OR 1.40 [95% CI 1.16–1.68]), shock (OR 1.51 [95% CI 1.14–1.99]), and presumed pulmonary oedema (OR 1.58 [95% CI 1.04–2.39]). Lower in-hospital mortality was associated with moderate anaemia (OR 0.87 for a decrease of 10 percentage points in haematocrit [95% CI 0.80–0.95]). Circulating parasite density was not associated with mortality (OR 1.02 for a 6-fold increase [95% CI 0.94–1.11]), so the pathological effects of parasitaemia appear to be mediated entirely by the downstream effects of sequestration. Treatment with an artemisinin derivative decreased mortality compared with quinine (OR 0.64 [95% CI 0.56–0.74]). These estimates were consistent across children and adults (mainly representing African and Asian patients, respectively). Using inverse probability weighting, transfusion was not estimated to be beneficial in children with admission haematocrit values between 15% and 25% (OR 0.99 [95% CI 0.97–1.02]). Except for the effects of artemisinin treatment and transfusion, causal interpretations of these estimates could be biased by unmeasured confounding from severe bacterial sepsis, immunity, and duration of illness.

**Conclusion:**

These data suggest that moderate anaemia is associated with a reduced risk of death in severe falciparum malaria. This is possibly a direct causal association. The severe anaemia threshold criteria for a definition of severe falciparum malaria should be reconsidered.

## Abstract 2

Nicholas White and colleagues study physiological and clinical pathways associated with mortality in severe Plasmodium falciparum malaria.

## Author summary

**Why was this study done?:**

- Severe falciparum malaria is a medical emergency with high patient fatality, even following treatment with parenteral artesunate.
- No adjunctive treatments have proved effective despite considerable research, except renal replacement therapy in patients with acute renal failure.
- Understanding the causal contributions of the different key patient variables at presentation could improve the definition of severe falciparum malaria, patient triage, and the prioritisation of future trials aimed to assess novel treatments.

**What did the researchers do and find?:**

- We pooled data from 9,040 case records from prospective studies in patients with strictly defined severe falciparum malaria conducted across Asia and Africa.
- We constructed causal diagrams based on expert knowledge showing what we consider to be the best description of the causal pathways leading to death in severe malaria.
- This model was used to obtain generalisable estimates of the causal contributions to a fatal outcome of coma and increases in base deficit, shock, renal impairment, seizures, presumed pulmonary oedema, and anaemia.
- We found that patients with lower haematocrits were at a lower risk of death and that there was no strong evidence for a beneficial effect of transfusion in moderately anaemic children (15%–25% haematocrit).

**What do these findings mean?:**

- In the context of severe falciparum malaria, moderate anaemia may protect against death, and a conservative approach to blood transfusion might be warranted.
- The current haemoglobin threshold of 5 g/dL used to define severe malarial anaemia may be too high and should be reconsidered.
- In order to refute or confirm these findings, randomised studies are needed to assess optimal transfusion thresholds.

## Introduction

Severe falciparum malaria remains an important cause of premature death in malaria-endemic countries [1,2]. The onset of life-threatening complications can be rapid, particularly in children, leaving little or no time to reach hospital [3]. The majority of in-hospital deaths occur within the first 24 hours following admission [4]. Patient fatality following treatment with parenteral artesunate is significantly lower than with quinine but still ranges between 10% and 30%, depending on the number and severity of complications present on admission [5–7].

Assessing the predictive value of admission ‘phenotypes’, as defined by clinical and laboratory covariates, on the outcome of severe malaria (i.e., risk factors) may improve the recognition of patients who are likely to deteriorate and thus help identify those who would benefit most from escalation of care [1]. This does not require ascribing causality. However, determining causal relationships between measurable admission variables and outcome does provide a rational basis for optimising treatment and evaluating life-saving adjuvant interventions. This is an important consideration, as many adjuvant therapies have been evaluated in severe malaria, often without a clear rationale. Apart from renal replacement therapy, to date none of these have been proved effective, and several were shown to be harmful [8–11]. From a pragmatic perspective, the use of causal inference tools allows for the identification of key confounders and possible selection bias [12]. Subsequent risk factors will be more generalisable than those generated from a causal inference-naive prediction model.

In the absence of randomisation, estimating the direction and magnitude of a causal contribution relies on multiple assumptions that are nonstatistical in nature [13]. The past 3 decades have seen considerable advances in the field of causal inference, and these have had major implications for epidemiology research. Counterfactual reasoning is the dominant framework. This attempts to estimate contrasts of potential outcomes between groups who did receive and did not receive an exposure of interest [14]. Causal diagrams, represented by directed acyclic graphs (DAGs), are the key algebraic tool. Within the area of causal inference in epidemiology, there is debate as to which experimental conditions allow for the valid estimation of causal effects [15,16]. ‘No causation without manipulation’ posits that causation can only be derived in the presence of well-defined, humanly possible interventions [17]. This implies that the main goal of causal inference is to mimic idealised—even if practically unfeasible—randomised experiments [18]. However, this precludes studying the causal effect of many health states, for which multiple different interventions exist with different average outcomes, or immutable states such as sex or race [13].

In this work, we take a pragmatic approach to the important question, ‘Which clinical or pathological features of severe falciparum malaria cause death, and by how much?’ We attempt to estimate the causal contributions to mortality of key, routinely measured, clinical variables in severe falciparum malaria. Our approach is based on the structural model causal inference framework [13], underpinned by mechanistic understanding of the causal and temporal processes in severe malaria. We constructed template causal diagrams of the main causal pathways in severe malaria. These outlined our hypotheses concerning the relationships between clinical and laboratory covariates measured before starting treatment for severe malaria and mortality, and so they were used to determine the set of adjustment variables necessary to remove confounding bias. The fundamental biological assumption is that the major process leading to death in severe falciparum malaria—an acute and life-threatening syndrome—is the extensive sequestration of parasitised erythrocytes in the microvasculature leading to vital organ dysfunction [1,8,19]. The resulting causal diagrams were used as a basis for principled regression models, with in-hospital death as the predicted outcome, using one of the largest prospectively collected pooled datasets of patients with severe falciparum malaria, comprising 9,040 children and adults from Africa and Southeast Asia.

## Methods

### Causal inference framework

We used a structural model causal inference approach [13]. All our causal assumptions were formalised in a causal diagram (DAG). Clinical and laboratory covariates included in the DAG were chosen pragmatically and based on previously described insights into the mechanism of disease. The final DAG is displayed in Fig 3 and can be accessed online on the DAGitty website (http://dagitty.net/development/dags.html?id=HJ1OW3). DAGitty is an online platform for the analysis of causal diagrams and implements causal calculus (d-separation rules). Conditional on the causal diagram under consideration, this determines the necessary adjustment sets to allow for unbiased estimation of the total or direct causal effects on the outcome variable. We make the distinction between direct effects and total effects. The direct effect is the effect of the exposure (e.g., anaemia) on death at a fixed level of the mediator (e.g., for a fixed degree of acidosis). This is termed a controlled direct effect in the causal inference literature. The total effect includes indirect effects via mediating variables (e.g., acidosis).

#### DAG describing causal pathways in severe falciparum malaria

We based our DAG (Fig 3) on the general assumption that the onset and severity of severe falciparum malaria are a function of the sequestered pathogen (parasite) load, which is the key process leading to vital organ dysfunction and potentially resulting in a fatal outcome. Therefore, in the DAG model describing the sequential pathogenic mechanisms that result in severe falciparum malaria, observed parasite biomass (circulating parasite densities measured in a peripheral blood smear) plays a central role, as this may be considered a proxy for the more important unobserved parasite biomass (sequestered parasites). Although we believe this DAG represents important relationships between clinical and laboratory covariates measured in patients with severe malaria, the pathogenic mechanisms that govern interindividual variation in disease severity and organ failure at the time of hospital admission in nonimmune hosts with severe falciparum malaria remain poorly understood.

Fig 3 shows the posited direct relationships between circulating parasitaemia (observed), sequestered parasitaemia (unobserved), the main clinical and laboratory determinants of the severe falciparum malaria classification, and death. Circulating parasitaemia is also a determinant of this classification. Implicit stratification resulting from including only patients who are classified as having severe malaria is represented by the vertex ‘Included in study’. Eligibility criteria for patients in the final dataset were based on WHO criteria for severe malaria, with minor variations as stated in the original publications and summarised in S1 Table. The time dimension in this DAG, which is not visualised explicitly, is very important. For example, the antimalarial treatment received, shown by the vertex ‘Drug’, affects parasitaemia directly but only after it has been given, which is why there is no arrow from ‘Drug’ to ‘Parasitaemia’.

The goal of this analysis was to determine the individual total and direct causal effects on mortality (represented by the vertex ‘Death’) of anaemia (i.e., haematocrit), acute kidney injury (AKI, as measured by blood urea nitrogen), coma (cerebral malaria), seizures, shock, acidosis (base deficit), pulmonary oedema (clinical diagnosis from respiratory distress and abnormal chest auscultation), and circulating parasitaemia (proportion of red blood cells that are parasitised measured in a peripheral blood film using light microscopy).

The important unmeasured variables are ‘Sepsis’ and ‘Lactate’ (missing in 88% of cases, see section Statistical analysis). A significant proportion of unmeasured bacterial sepsis caused by the malarial infection would result in confounding for all exposure–outcome relationships. Bacteraemia, particularly with enteropathogens, is common in children diagnosed with severe malaria, but it is uncommon in adults. Although severe malaria does predispose to sepsis, many cases in children in endemic areas diagnosed as severe malaria are likely to represent primary septicaemia with incidental malaria [8]. In the following, we have assumed that bacterial sepsis caused by the malarial infection (predisposition) results in only minor confounding bias. Using d-separation rules (for a recent review, see [13]) assuming the DAG in Fig 3 is correct, the minimal adjustments that allow for unbiased estimates of the total or direct causal effects are as follows:

- The total effect of anaemia on death cannot be estimated; however, the direct effect can be estimated with adjustments required for pulmonary oedema, AKI, shock, acidosis, hypoglycaemia, seizures, and coma.
- The direct effect of coma on death can be estimated with adjustments required for pulmonary oedema, AKI, shock, acidosis, hypoglycaemia, anaemia, and seizures (mediator).
- The total (which is also the direct effect) of acidosis on death can be estimated with adjustments required for AKI, anaemia, coma, seizures, hypoglycaemia, pulmonary oedema, and shock.
- The direct effect of shock on death can be estimated with adjustments required for AKI, acidosis (mediator), anaemia, coma, hypoglycaemia, pulmonary oedema, and seizures.
- The direct effect of AKI on death can be estimated with adjustments required for acidosis (mediator), anaemia, coma, seizures, hypoglycaemia, pulmonary oedema, and shock.
- The total (which is also the direct effect) of pulmonary oedema in death can be estimated with adjustments required for AKI, acidosis, anaemia, coma, hypoglycaemia, seizures, parasitaemia, and shock.
- The direct effect of parasitaemia on death can be estimated with adjustments required for AKI, acidosis, anaemia, coma, hypoglycaemia, pulmonary oedema, seizures, and shock.
- The total (which is also the direct effect) of seizures on death can be estimated with adjustments required for AKI, acidosis, anaemia, coma, hypoglycaemia, pulmonary oedema, and shock.

Any resulting association between these factors and mortality may then be interpreted as causal. This causal interpretation relies on three key assumptions, known as conditional exchangeability (no unmeasured confounding), consistency, and positivity. Consistency means that the probability of the outcome is the same whether the value of a variable was simply observed or was intervened upon. For example, in this context, consistency implies that the probabilities of death in a patient with 35% haematocrit on admission and in an identical patient but with 10% haematocrit on admission who received a blood transfusion leading to 35% haematocrit are equal (in the highly idealised scenario in which blood transfusions happen instantaneously). Positivity means that every value of the exposure variable (e.g., haematocrit) has a nonzero probability of occurring in all patient strata.

In the statistical analyses, we adjusted for antimalarial treatment (‘Drug’) in order to reduce the variance in the final estimates of the causal effects. We also adjusted for age and access to care (using the proxies study site and country) which explains large differences between trials and countries (especially between African and Asian sites).

#### ‘Well-definedness’ of causal questions represented in the DAG

A reasonable criticism of the causal DAG shown in Fig 3 is that the arrows from the exposure variables (shown in green) to the outcome variable (‘Death’: red) do not represent well-defined causal questions [18]. This corresponds to the interventionist perspective on the potential outcomes framework, which has been labelled the ‘restricted potential outcomes approach’ [15].

We argue that the exposure variables in Fig 3 do correspond to well-defined exposures from an interventionist perspective—i.e., there are well-defined interventions. We focus this methodological reflection on haematocrit (anaemia), with analogous arguments for renal failure, acidosis, coma, seizures, and pulmonary oedema. In a population of interest, steady-state haemoglobin concentrations can be thought of as randomly assigned, corresponding to differences in host genetics (e.g., haemoglobinopathies), sex, age, and environmental influences (diet, recent haemolytic events such as malaria, the bone marrow dysfunction associated with malaria, intestinal helminth coinfections, geographical location, and access to healthcare). When considering admission haematocrits in the context of severe-malaria clinical trials, additional factors are important: host immunity, host splenic function, host–parasite interactions, and parasite genetics (all potentially affecting parasite multiplication rates and pathogen load). Note that some of these do confound the relationship with survival and should therefore be adjusted for. However, the non-confounding factors (some host genetics, age, sex, diet, recent haemolysis, bone marrow dysfunction, coinfection) are likely to determine the majority of the variation in haematocrits observed at presentation. These factors are complex, but there is no reason to believe that their overall effect—changing the admission haematocrit—should not be approximately equivalent to the intervention of blood transfusion (or the hypothetical reverse intervention of removing blood). Therefore, we argue that the contribution of anaemia on mortality corresponds to a well-defined (from an interventionist perspective) causal question.

### Datasets

We included in this analysis all randomised trials and observational studies conducted or coordinated by the Mahidol Oxford Tropical Medicine Research Unit, Bangkok, Thailand, and the Oxford University Clinical Research Unit, Hospital for Tropical Disease, Ho Chi Minh City, Vietnam, between 1980 and 2016. The pooled dataset was made up of records for 9,040 patients (5,446 children less than 12 years old and 96 adults from sub-Saharan Africa, and 189 children and 3,309 adults from Southeast Asia). Each study included in this pooled analysis was approved by a local or national ethics committee and by the Oxford tropical research ethics committee. All patients, or their attendant relative or guardian, provided written informed consent. For further details, see the references provided below.

- ‘Core Malaria’, Thailand: observational and treatment studies of severe-malaria patients enrolled in hospital-based studies conducted between 1980 and 2016 in Chantaburi, Kanchanaburi, and Tak provinces (Thailand) (*n* = 683) [20,21].
- ‘Core Malaria’, Bangladesh: observational and treatment studies of patients with severe malaria enrolled at the Chittagong Medical College Hospital, Chittagong, Bangladesh, carried out between 2003 and 2016 (*n* = 424) [22–24].
- The quinine versus chloroquine trial in The Gambia (QC): a randomised controlled trial of quinine versus chloroquine in children with severe malaria enrolled at the Royal Victoria Hospital and the United Kingdom Medical Research Council laboratories and wards, The Gambia, from September to October 1988 (*n* = 48) [25].
- The artemether versus artesunate trial in Vietnam (AAV): a randomised controlled trial of artesunate versus artemether in Vietnamese adults with severe falciparum malaria conducted at the Hospital for Tropical diseases, Ho Chi Minh City, Vietnam, between May 1996 and June 2003 (*n* = 370) [26].
- The artemether versus quinine trial in Vietnam (AQ): a randomised controlled trial of parenteral artemether versus quinine in Vietnamese adults with severe falciparum malaria conducted at the Hospital for Tropical diseases, Ho Chi Minh City, Vietnam, between May 1991 and January 1996 (*n* = 560) [27].
- The South East Asian multinational quinine versus artesunate malaria trial (SEAQUAMAT): a multicentre, open-label, randomised trial of artesunate versus quinine for treatment of severe falciparum malaria conducted in Papua, Myanmar, Bangladesh, and India between June 2003 and May 2005 (*n* = 1,461) [5].
- The Africa multinational quinine versus artesunate in severe-malaria trial (AQUAMAT): a multicentre, open-label, randomised controlled trial of artesunate versus quinine in the treatment of severe falciparum malaria in African children conducted in 11 sites located in nine countries between October 3, 2005, and July 14, 2010 (*n* = 5,494) [28].

Details of the study populations and methods used in these studies can be found in the individual publications (see Table 1 for a brief overview). Inclusion criteria varied between some studies, as shown in S1 Table. Data merging and cleaning were performed using Stata version 14.0.

**Table 1:** Key characteristics and published references for the pooled dataset of severe-malaria patients.

Study name	Study countries	Number of patients	In-hospital mortality (%)	Median age, years (range)	Parenteral antimalarial treatments	References
**Core Malaria**	Thailand Bangladesh	1,107	22	26 (1–80)	Artemether Artesunate Quinine	[22] [21] [24] [20] [23]
**AAV**	Vietnam	370	10	32 (15–77)	Artemether Artesunate	[26]
**AQ**	Vietnam	560	15	30 (15–79)	Artemether Quinine	[27]
**QC**	The Gambia	48	17	4.5 (1–10)	Quinine Chloroquine	[25]
**SEAQUAMAT**	Bangladesh Myanmar India Indonesia	1,461	19	25 (2–87)	Artesunate Quinine	[5]
**AQUAMAT**	Mozambique Ghana Kenya The Gambia Nigeria Tanzania Uganda Rwanda DRC	5,494	10	2 (0–78)	Artesunate Quinine	[28]
**Overall**	-	**9,040**	**13**	**4** (0–87)		

AAV, AQ, QC, SEAQUAMAT, and AQUAMAT were randomised controlled trials.

Abbreviation: AAV, artemether versus artesunate trial in Vietnam; AQ, artemether versus quinine trial in Vietnam; AQUAMAT, Africa multinational quinine versus artesunate in severe-malaria trial; DRC, Democratic Republic of Congo; QC, quinine versus chloroquine trial in The Gambia; SEAQUAMAT, South East Asian multinational quinine versus artesunate malaria trial.

### Statistical analysis

All statistical analyses were done in R version 3.4.3 (November 30, 2017). All analyses are fully reproducible and publicly available as an RMarkdown notebook on GitHub, which can be found at https://github.com/Stije/SevereMalariaAnalysis. This analysis of all prospectively studied patients was not prespecified using a published statistical analysis plan, and all analyses as such should be considered not prespecified.

### Multiple imputation

In the pooled dataset, several variables were missing in a significant proportion of records. Coma status was missing from 229 records (3%), haematocrit from 870 (10%), parasite density from 1,882 (21%), base deficit from 2,375 (26%), plasma bicarbonate from 3,679 (41%), respiratory rate from 369 (4%), venous blood or plasma lactate from 7,423 (82%), blood urea nitrogen from 1,544 (17%), plasma creatinine from 7,242 (80%), transfusion information from 2,342 (22%), and information on hypoglycaemia from 115 records (1%).

Imputation of missing variables was done using known mechanistic relationships with other recorded variables and was done sequentially, processing from the most-informative recorded variable to the least informative.

- We used the auxiliary variables bicarbonate, lactate, and respiratory rate to impute missing base deficit values. For base deficit, this was done with Gaussian error linear models, first using bicarbonate, and then, if that value was also missing, we used lactate and then finally respiratory rate for the remaining missing values. Remaining missing values were then modelled from the marginal age-adjusted distributions.
- We used plasma creatinine to impute missing blood urea nitrogen values. Imputation for missing blood urea nitrogen values was also done with a Gaussian linear model on the log scale, with creatinine used as the predictive variable. Remaining missing values were imputed from the marginal distribution.
- Missing pulmonary oedema values were imputed from respiratory rate with Gaussian error.
- Missing haematocrit values were imputed from age with Gaussian error.
- Missing parasitaemia values were imputed from age on the log scale with Gaussian error.

Every imputation model had random-effect terms for country and study. A total of 100 imputed datasets were created for the final analysis. The full imputation code is available online at https://github.com/Stije/SevereMalariaAnalysis.

#### Regression modelling

Patient data are denoted ${\left\{y_{i},\;x_{i}\right\}}_{i=1}^{N}$, where *N* = 9,040, *y*_*i*_ is the outcome by end of hospitalisation (died/survived), and *x*_*i*_ is the set of explanatory variables as given above: haematocrit, coma, blood urea nitrogen, base deficit, pulmonary oedema, hypoglycaemia, seizures, shock, parasitaemia, age, treatment drug, study ID, country, and study site.

The estimation of the causal relationship between the set of baseline covariates ***z*** ∈ ‘haematocrit, coma, base deficit, blood urea nitrogen, pulmonary oedema, seizures’, and survival *y*_*i*_ from severe-malaria clinical trial data was done as a two-stage process:

In the first stage, we did the following: For each imputed dataset *j* = 1…100, we fitted a mixed-effects logistic regression model, whereby *y*_*i*_ ~ *L*(***x***_***i***_|***β***), which characterised the relationship between survival *y*_*i*_ and the observed covariates ***x***_***i***_. Patients were indexed by *i*. This model adjusts for confounders and blocks ‘back-door paths’ conditional on the structural causal model shown in Fig 3. Included in ***x*** as fixed effects is the set of necessary adjustment variables. One exception is ‘Access to care’, which is modelled as an independent random effect using two proxy variables:

- study
- country

This model fit estimates the set of parameters ${\hat{\beta }}_{j}$. Parameter estimation was done using the R package ‘lme4’ [29].

In a second stage, the sets of parameters ${\left\{{\hat{\beta }}_{j}\right\}}_{j=1}^{100}$ were then combined using Rubin’s rules in order to estimate overall fixed and random effects (functions ‘modelFixedEff’, ‘modelRandEffStats’ in ‘merTools’ [30]) and obtain a global estimate $\hat{\beta }$.

The global estimate $\hat{\beta }$ was then used to derive adjusted odds ratios along with CIs for the six factors of interest.

We performed a sensitivity analysis using only the patient records from studies done in Asia (*n* = 3,498). This analysis used 100 imputed datasets, with the same imputation procedures as above. We believe that data from Asian adults will not be significantly confounded by the presence of sepsis, as this has been investigated prospectively, and the estimated prevalence in adult severe falciparum malaria at presentation is less than 2%. This is based on observations from the AQ and AAV studies done in Vietnam, whereby admission blood cultures were performed systematically. In the AQ study, eight patients had positive blood cultures out of 560 (1.4%), and in the AAV study, six out of 220 were positive, but two of these were coagulase-negative staphylococci, which were likely contaminants, giving a global estimate of 1.8% (the remaining 150 records in the AAV study could not be matched).

We performed an additional sensitivity analysis only using the patient records for which there were complete data (*n* = 5,542). For many of the missing key variables of interest (such as base deficit), values will not be ‘missing completely at random’, but it can be assumed that they are ‘missing at random’ (e.g., the missing status will be independent of the value of the covariate). In most cases, data are missing because the necessary tests or machines were not available for that study. Therefore, a sensitivity analysis only using patient records with full data (for the variables of interest) should not introduce any major biases but will have reduced power.

A mediation analysis was performed to see whether blood transfusion explains the protective effect of lower haematocrit values. As shown by the DAG in panel A of Fig 5, to reduce selection bias, we stratified by outcome at 4 hours, only selecting those patients who survived more than 4 hours. The model then adjusted for transfusion in addition to the set of adjustment covariates included in the main model.

#### Inverse probability of transfusion weighting using propensity scores

In order to obtain marginal causal estimates for the effect of transfusion on death in children with admission haematocrits within a ‘moderate anaemia range’ of 15%–25%, we used inverse probability weighting (IPW) with propensity scores. The range of 15%–25% haematocrit was chosen, as it is above the AQUAMAT trial threshold for transfusion (15% haematocrit). Below 25% haematocrit, the estimated weights were all less than 10. The sample size was not large enough for an equivalent analysis in adults, as only the AQ and AAV studies reliably recorded transfusion status.

The inverse probability weights were computed using the R package ‘ipw’ [31]. This computed stabilised weights for each imputed dataset, whereby the numerator of each weight was the marginal probability of the intervention received (either transfused or not transfused), and the denominator was the individual probability of the intervention (transfused or not transfused) estimated using a fixed-effects logistic regression model with the following independent predictors: the site country, admission haematocrit, presence of convulsions, base deficit (mEq/L), log base 2 blood urea nitrogen (mmol/L), respiratory rate (breaths per minute), and age (years).

This analysis used the data from the AQUAMAT study to estimate the effect of transfusion on death. Patients enrolled in the Democratic Republic of Congo were excluded, as almost all of these patients were given a blood transfusion (only 16 out of 422 did not receive one). The final analysis included 2,214 with baseline haematocrits between 15% and 25%, of which 1,083 were transfused, and 1,131 were not transfused. The effect of transfusion on death in the IPW population was estimated with a robust sandwich estimator using the function ‘svyglm’ from the R package ‘survey’ [32]. Balance of key covariates was assessed by calculating absolute standardised differences between the transfused and not-transfused groups [33].

## Results

### Pooled data and patient characteristics

We pooled individual patient data from all clinical trials in severe falciparum malaria run by or coordinated by the Mahidol Oxford Tropical Medicine Research Unit based in Bangkok, Thailand, and the Oxford University Clinical Research Unit based in Ho Chi Minh City, Vietnam, conducted between 1980 and 2015. The final pooled dataset included records from 9,040 individual patients enrolled in four large randomised controlled trials in severe malaria and a series of observational studies and one small randomised trial (quinine versus chloroquine) conducted in The Gambia. Table 1 gives an overview and summary of the main characteristics of these studies, which span 15 different countries. The inclusion and exclusion criteria of these pooled clinical studies overlapped almost completely and are summarised per individual dataset in S1 Table. Overall mortality varied from 10% in the AQUAMAT (mainly African children) and AAV (Vietnamese adults) studies to 22% in the Core Malaria records (Thai and Bangladeshi adults). Patients from Southeast Asia contributed approximately 40% of the total pooled records.

### Exploratory data analysis

**Fig 1:**
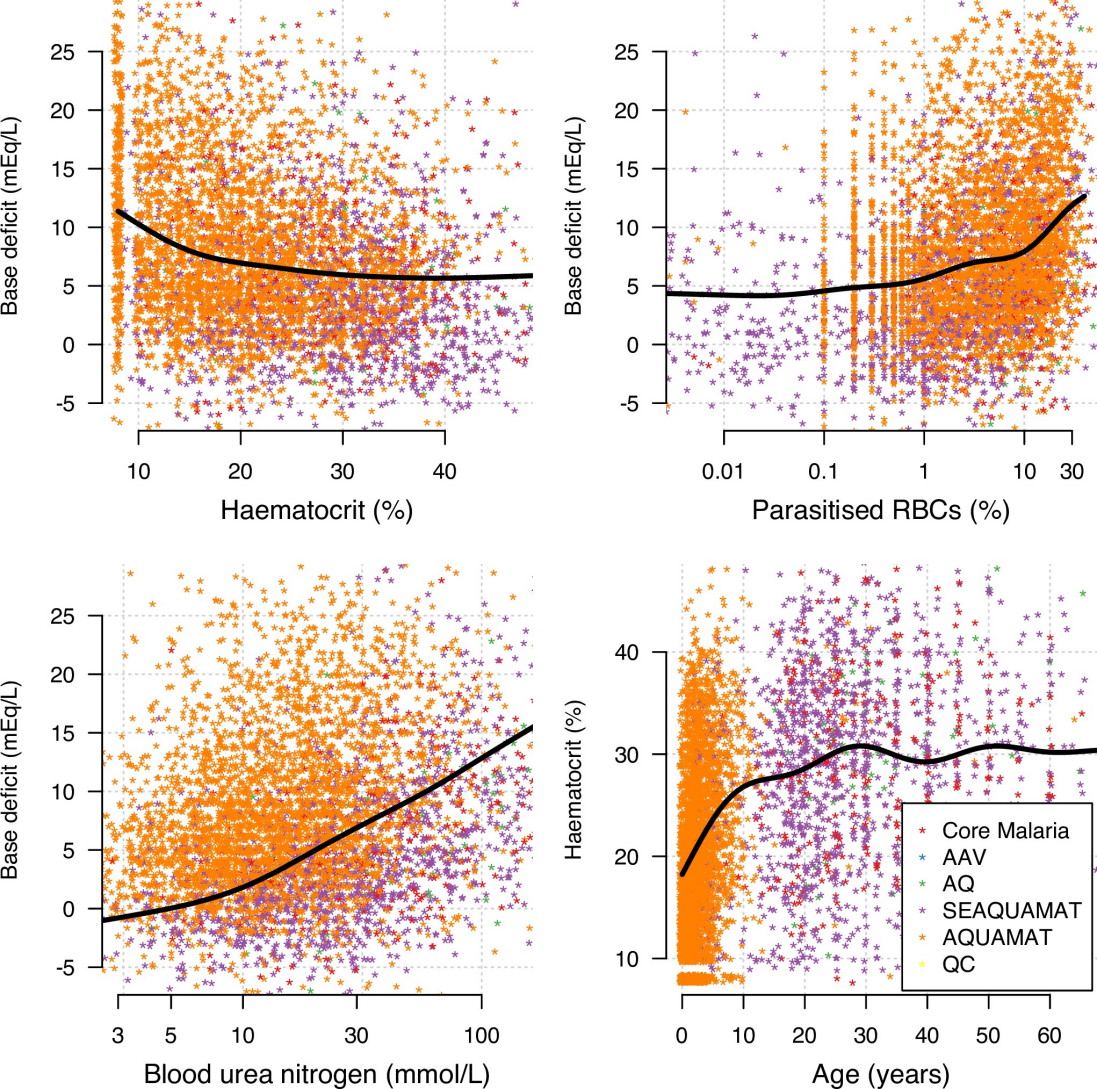
Scatterplots of observed associations between key patient covariates in the pooled severe-malaria dataset. Mixed-effects spline fits are shown by the thick black lines, estimated using generalised additive models, which include independent random-effect terms for both ‘country’ and ‘study’. No base deficit measurements were taken in the AAV or QC studies. Axes are scaled to contain 98% of the data (outliers were cut at the 1% and 99% quantiles). Uniform random jitter over the interval (–0.4, 0.4) was added to the recorded values of haematocrit, base deficit, blood urea nitrogen, and age. The x-axes for blood urea nitrogen and parasitised RBCs (%) are on a log_10_ scale. AAV, artemether versus artesunate trial in Vietnam; AQ, artemether versus quinine trial in Vietnam; AQUAMAT, Africa multinational quinine versus artesunate in severe-malaria trial; QC, quinine versus chloroquine trial in The Gambia; RBC, red blood cell; SEAQUAMAT, South East Asian multinational quinine versus artesunate malaria trial.

The considerable interdependencies between variables are highlighted in the bivariate scatter plots along with mixed-effects spline fits (Fig 1). Marked separation in age between the different studies is clearly shown in the lower-right panel, with a strong positive correlation between age and haematocrit in children. The majority of data on young children are from the AQUAMAT study.

**Fig 2:**
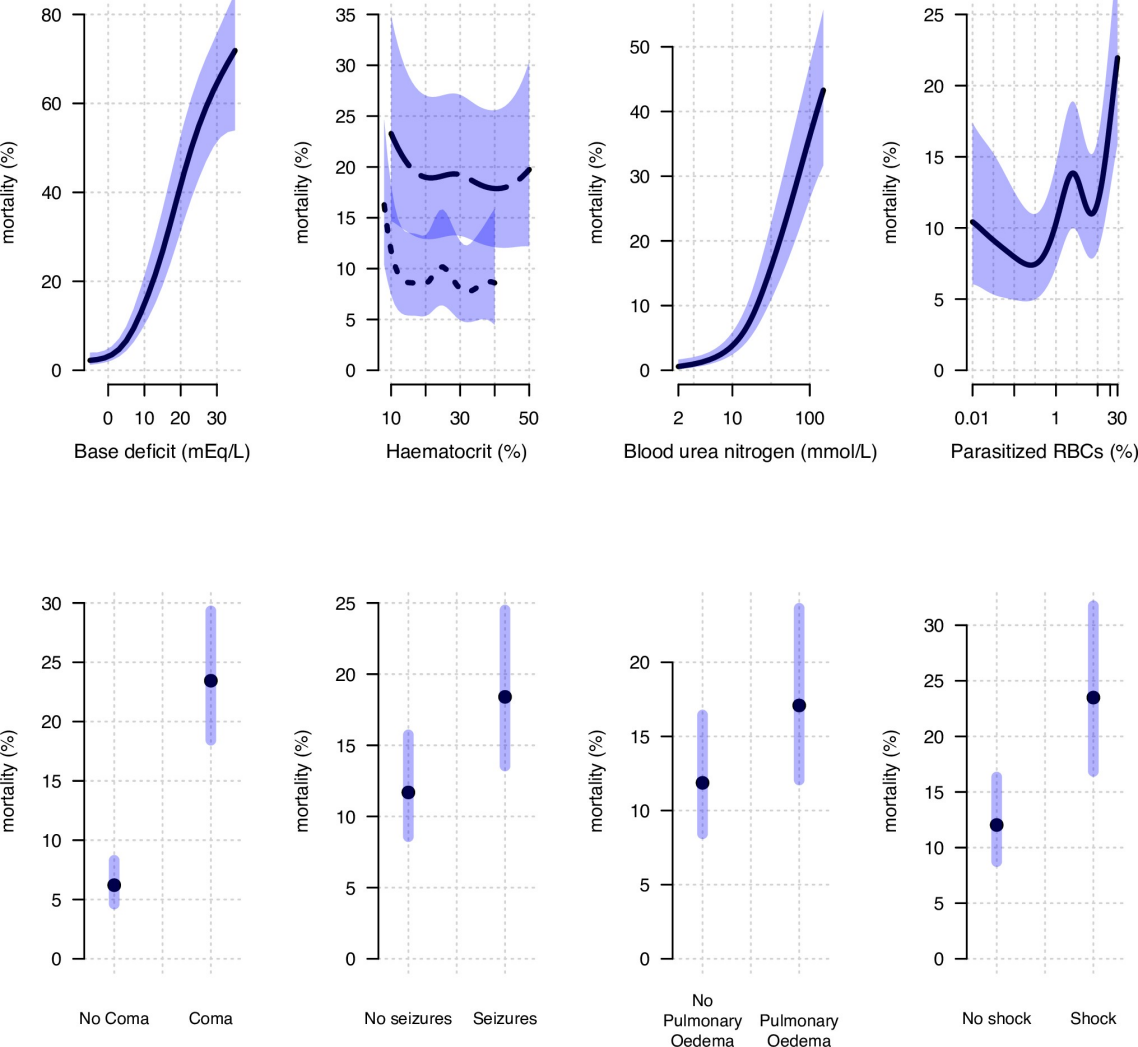
The unadjusted associations between in-hospital mortality from severe falciparum malaria and the eight clinical factors of interest. For the continuous variables (top row), the thick black lines (blue shaded areas) show the mean (95% CI) values from a mixed-effects logistic generalised additive model for which the independent random-effect terms were ‘study’ and ‘country’. The generalised additive models allow nonlinear relationships between the continuous covariates and mortality. The relationship between haematocrit and death is shown separately for children (≤12 years old) by the dotted line and for adults by the dashed line. Their ranges are taken to represent the 1%–99% quantile ranges for children and adults, respectively. For the binary variables (bottom row), the black dots (blue intervals) show the mean (95% CI) for a mixed-effects logistic regression model, with the same random-effect terms. The x-axes for blood urea nitrogen and percent of parasitised RBCs are on a log_10_ scale. RBC, red blood cell.

The estimated univariate regression models, adjusting only for intersite and intercountry variability and allowing for nonlinear relationships between the four continuous baseline characteristics of interest and in-hospital mortality, are shown in Fig 2. Base deficit (acidosis) and blood urea nitrogen (AKI) were strong univariate predictors of a fatal outcome with monotonic relationships to mortality, as described previously [7]. By contrast, excess mortality was observed across the range of admission haematocrits (95% of the data lie in the haematocrit interval between 8% and 44%). The association between admission haematocrit and death had a ‘U-shape’, whereby both low-admission (below 15%) and high-admission (above 35%) haematocrit values were associated with an increased risk of death (Fig 2). For the binary predictors, standard logistic regression models were used, again adjusting for site and country. Coma and pulmonary oedema were the strongest binary predictors of death, with unadjusted risk ratios of 4.38 (95% CI 3.75–5.12) and 4.54 (95% CI 3.07–6.71), respectively.

### Consequences of key assumptions in the diagram describing causal pathways in fatal falciparum malaria

**Fig 3:**
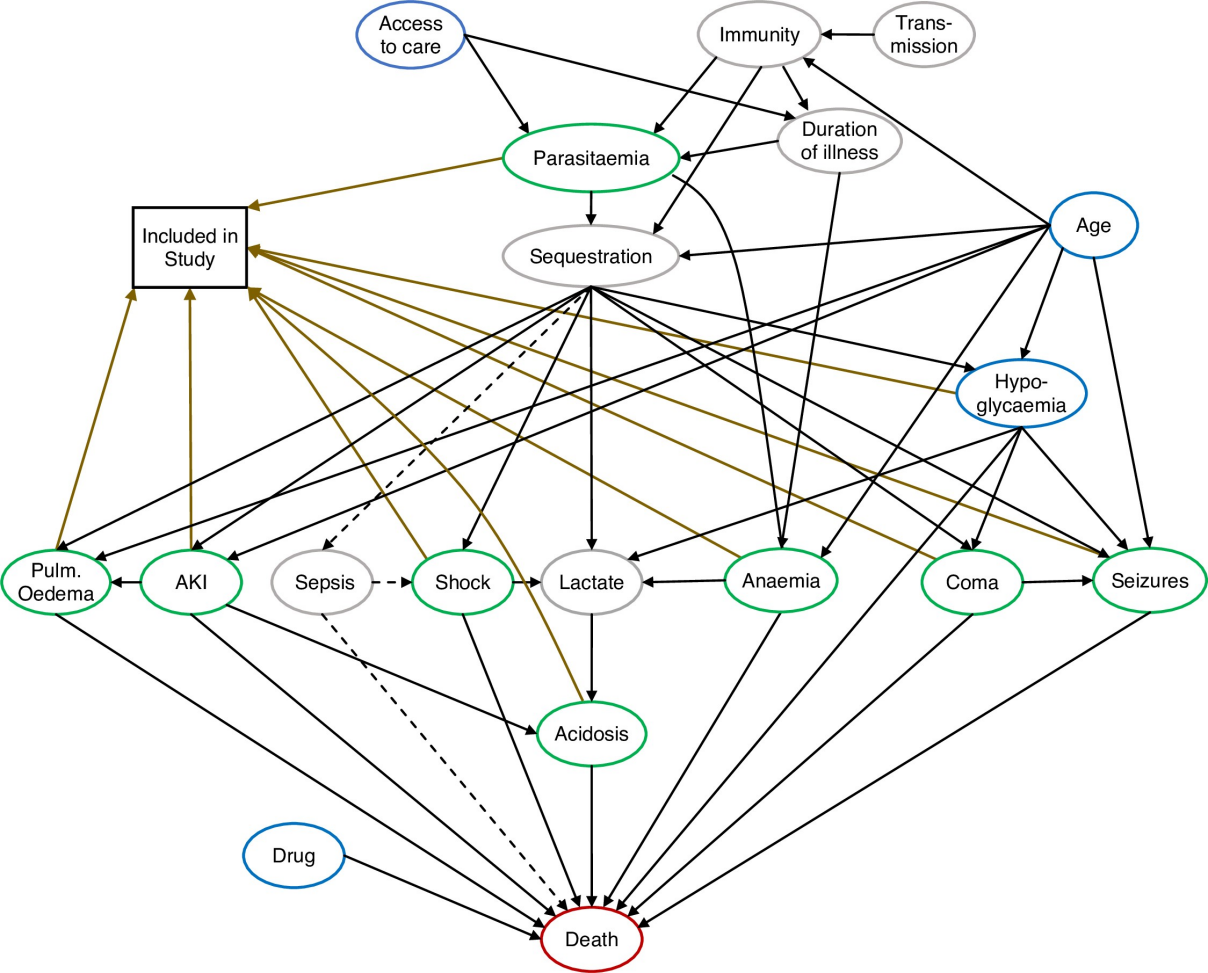
The DAG used to model the determinants of mortality in severe malaria. Each variable is represented by a vertex in the graph. Green vertices are the exposure variables of interest, and ‘Death’ (red) the outcome variable. Assumed direct (mechanistic) causal relationships between variables are shown by black arrows. Yellow arrows correspond to nonmechanistic relationships (study inclusion criteria). Grey vertices indicate unobserved or unmeasured variables; blue indicates observed variables (which are not exposure variables); the square vertex ‘Included in study’ denotes the de facto stratification resulting from the fact that data were available only on study patients fulfilling (to varying degrees) the predefined WHO severe-malaria criteria (see S1 Table). The causal pathway from ‘Sequestration’ via ‘Sepsis’ is shown with dashed lines, as it is ignored in the main analysis. If sepsis is thought to be an important contributor to death from falciparum malaria (as opposed to death from sepsis with concomitant parasitaemia) and this pathway is included, all exposure–outcome relationships will be confounded. ‘Lactate’ is shown as missing here, as it was not recorded for 88% of patients. The reproducible DAG that was used to assess necessary adjustment sets (see Methods) can be found at http://dagitty.net/development/dags.html?id=HJ1OW3. AKI, acute kidney injury; DAG, directed acyclic graph; Pulm. Oedema, pulmonary oedema.

The primary goal of this study was to estimate unbiased causal effects on the subsequent in-hospital mortality from severe falciparum malaria of the absolute admission values of haematocrit (anaemia), base deficit (acidosis), blood urea nitrogen (AKI), and circulating parasitaemia (percent of parasitised red blood cells) and the presence of coma, seizures, shock, and pulmonary oedema measured or observed on enrolment in these prospective studies. Antimalarial treatments have a major impact on outcome, and adequately powered randomised drug trials (with balanced randomisation) should remove any confounding effects and thus allow for direct causal interpretation of observed differences between groups. However, for baseline characteristics such as anaemia, randomisation is not possible. Mendelian randomisation (for example, using glucose-6-phosphate dehydrogenase [G6PD] deficiency, a causal genetic variant for lower haemoglobin concentrations in acute infections [34–36]) is also not possible, as G6PD deficiency may influence the outcome (survival) via other pathways [37]. Instead, we used a structural causal models framework based on the causal diagram (DAG) shown in Fig 3. This necessitates specification of all bivariate causal effects between variables of interest via expert knowledge [13]. This is a subjective exercise but has the benefit of displaying transparently all the underlying assumptions.

Our proposed causal diagram (Fig 3) posits that the extensive sequestration of parasitised erythrocytes in the microvasculature is the key pathological process leading to death from severe falciparum malaria [19]. This effect is dependent on immunity and age. Notably, age affects the pattern of vital organ dysfunction. In the main analysis, we assumed that sepsis due to secondary invasive bacterial infection was only a minor contributor to mortality from severe falciparum malaria. Severe malaria does predispose to concomitant bacteraemia, usually with gram-negative enteric pathogens, but few data are available on the prevalence of secondary invasive bacterial infections in patients with severe falciparum malaria versus primary sepsis with incidental malarial parasitaemia, and both are probably underreported in sites with limited blood-culture facilities [38,39]. In African children diagnosed with lethal malaria, sepsis was described to be common, but many of these cases had low-parasite-biomass infections [8], indicating that sepsis was the primary pathological process and not malaria. Under this assumption that sepsis is a minor confounder, it was possible to estimate the direct causal effects for all exposure variables of interest (Fig 3).

### Causal determinants of a fatal outcome in severe falciparum malaria

**Fig 4:**
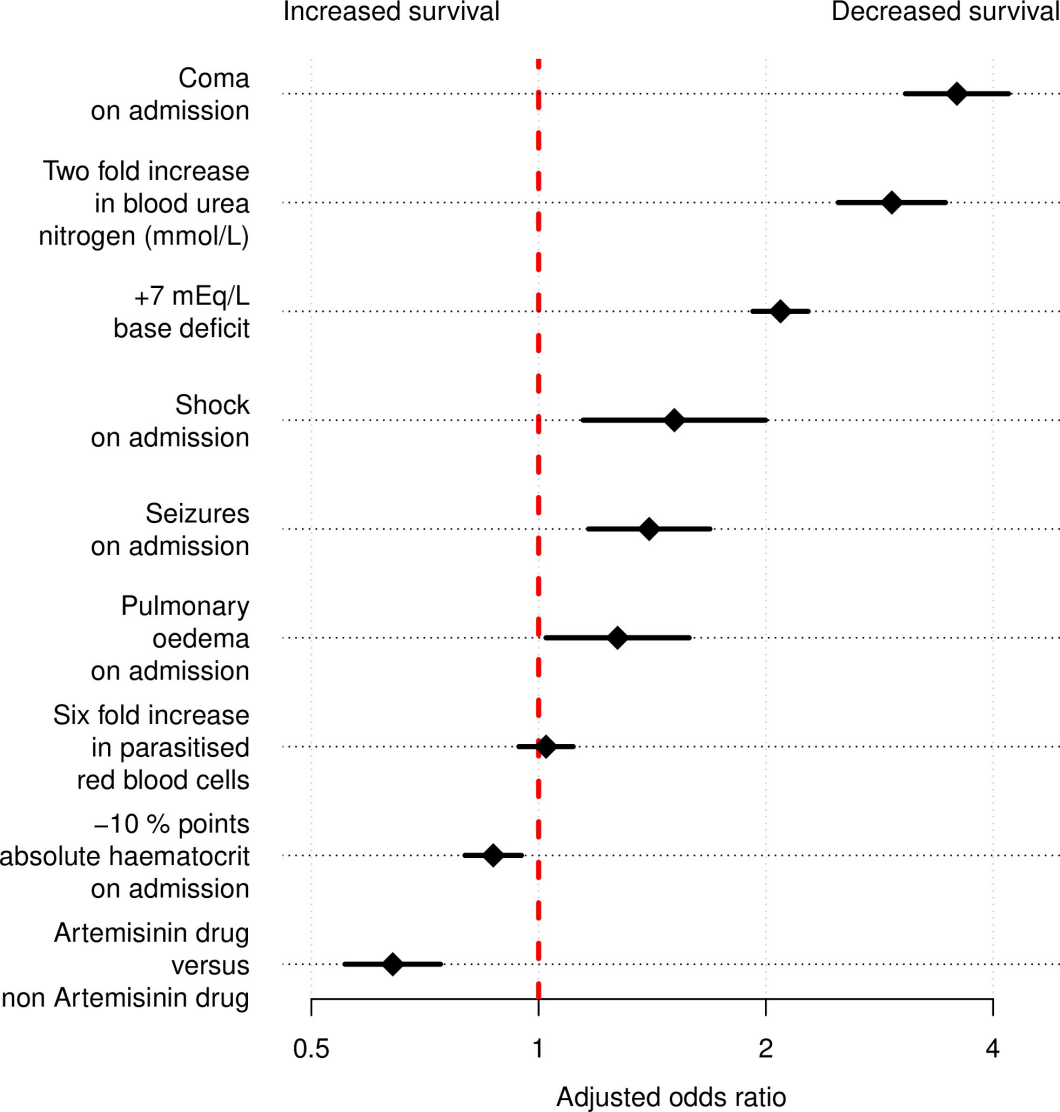
Estimated causal effects on mortality of main patient characteristics and treatment drug used. Adjusted odds ratios for death are shown on the log_2_ scale (values greater than 1 indicate a higher risk of death) and correspond to the combined results of a logistic regression model fitted to 100 independently imputed datasets. The scale of the unit changes was chosen to be approximately equal to 1 standard deviation of the population distribution (this is on a logarithmic scale for blood urea nitrogen and percent parasitised red blood cells).

Assuming a linear relationship between patient covariates and the log-odds of in-hospital mortality, we estimated that the adjusted mortality odds ratios varied from 0.87 (0.80–0.95), for an absolute decrease of 10 percentage points in haematocrit, to 3.59 (3.07–4.21), for the presence of coma. The full set of estimated causal effects are shown in Fig 4, with scaling units corresponding to 1 standard deviation in the population distribution. The proportion of parasitised red blood cells (parasitaemia) did not have a statistically significant direct effect on mortality, with an adjusted mortality odds ratio of 1.02 (0.94–1.11) for a 6-fold increase. The effect of parasitaemia was therefore mediated entirely by the downstream effects of sequestration, as shown in Fig 3. Treatment with an artemisinin derivative (artesunate or artemether) versus treatment with quinine or chloroquine (only 28 patients in the QC study received chloroquine) had an adjusted odds ratio for a fatal outcome of 0.64 (0.56–0.74). For simplicity, we have not included in Fig 3 the relevant arrows for the estimation of the causal effect of antimalarial treatment, but this estimate is likely to be valid, as either the treatment assignment of an artemisinin derivative versus quinine was randomised (AQUAMAT, AQ, SEAQUAMAT), or it was study dependent and the regression model adjusts for study. Therefore, under an additional assumption of no unknown confounders for treatment assignment in the observational studies, and no systematic bias in patients included in the AAV and QC study (these patients only received artemisinin derivatives and only non-artemisinin drugs, respectively), this adjusted odds ratio can also be interpreted as causal.

We performed two subgroup analyses with data only from Africa and only from Asia, respectively (S1 Fig). Overall, the results are consistent across the two settings (children versus adults and high versus low transmission, respectively). The subgroup analysis with only patients from Asia provides a sensitivity analysis with respect to the assumption of no significant confounding from bacterial sepsis (see justification in Methods). These results were also consistent with the results from the sensitivity analysis, which used only patient records with no missing variables (see online GitHub repository).

Under our causal model assumptions, ‘moderate anaemia’ was protective against mortality: a lower absolute admission haematocrit of 10 percentage points corresponded to a mortality odds ratio of 0.87 (95% CI 0.80–0.95). This assumes a linear relationship between haematocrit and the log-odds of death, which must be incorrect for extremely low haematocrit values. A sensitivity analysis removing patients with admission haematocrits less than or equal to 9% (a haemoglobin of approximately 3 g/dL) estimated a stronger protective effect, with an odds ratio of 0.83 (95% CI 0.75–0.91).

### Evaluation of the direct causal effect of moderate anaemia on outcome

**Fig 5:**
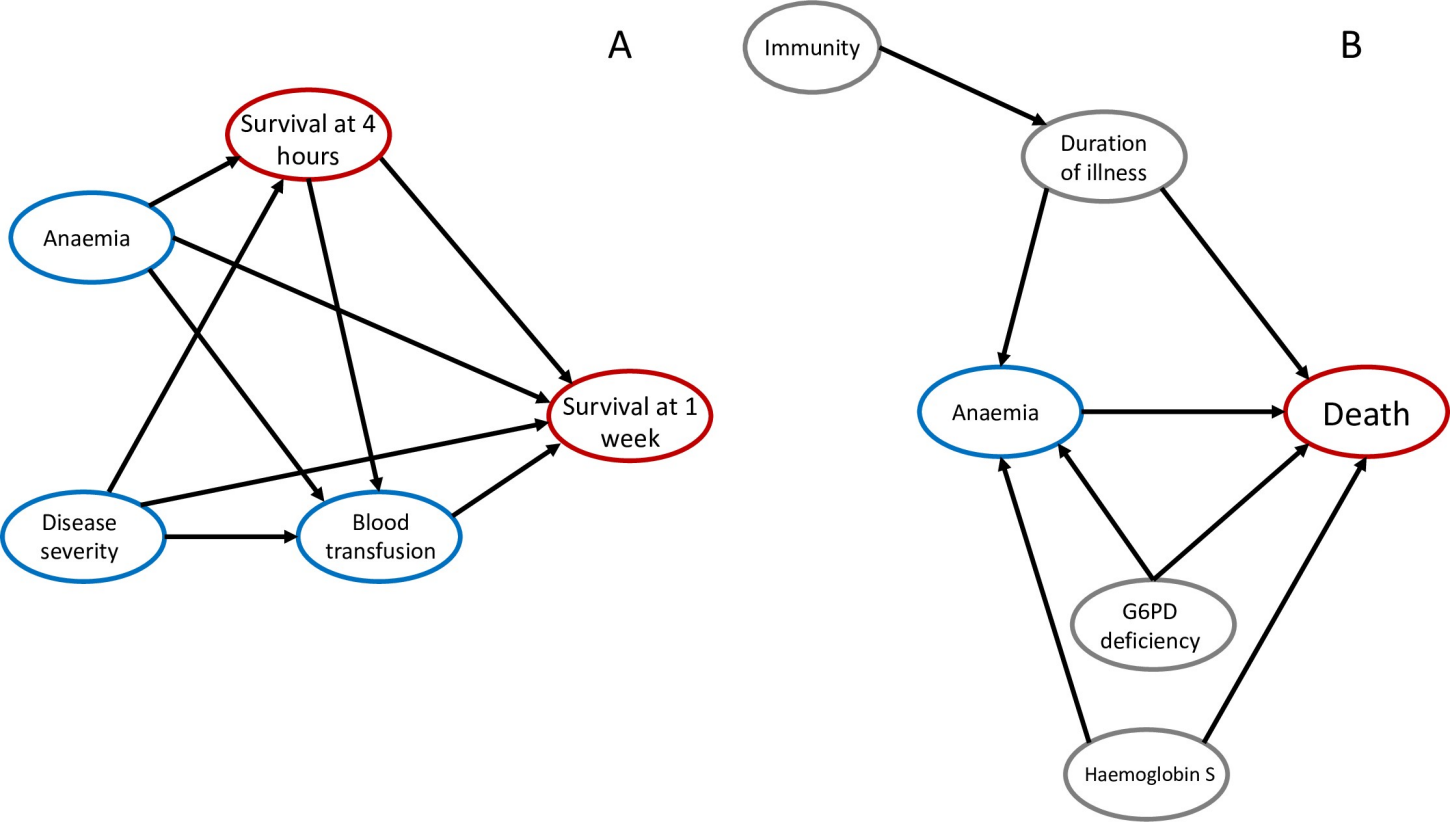
Mediation analysis and possible confounders for the protective effect of moderate anaemia on mortality in severe malaria. (A) One explanation for the apparent protective effect of moderate anaemia on fatal outcomes in severe falciparum malaria might be that blood transfusions given to anaemic patients are independently beneficial. The probability of receiving a blood transfusion is affected by the presence and severity of anaemia at baseline and also depends on blood availability and local treatment guidelines and practices. This DAG shows the proposed causal relationships between admission covariates, blood transfusion status, and in-hospital mortality, stratified by the survival outcome at 4 hours from admission (square vertex). ‘Disease severity’ is a simplified representation of the complex web of interactions shown in Fig 3. In particular, we note that ‘Blood transfusion’ is a ‘collider’ between ‘Anaemia’ and ‘Survival at 1 week’, thereby introducing selection bias. (B) Possible unmeasured variables confounding the relationship between anaemia and fatal outcome. Grey vertices are unmeasured genetic variables such as G6PD deficiency and haemoglobinopathies, which both cause anaemia directly and are thought to protect against death from severe malaria, or unobserved variables related to underlying immunity to malaria, which has complex effects both protecting against death and potentially causing prolonged illness and more red cell loss [1]. DAG, directed acyclic graph; G6PD, glucose-6-phosphate dehydrogenase.

Blood transfusion could mediate the relationship between anaemia and death. The apparent protective effect of lower haematocrits in severe malaria could result from the beneficial effect provided by blood transfusions given to patients presenting with haematocrits below a certain threshold (this varied by study and site but typically ranged from 15% to 20%). However, a naive mediation analysis via a simple adjustment for transfusion status results in selection (‘collider’) bias [40]. This is because of the interaction between time to death and blood transfusion, as shown in panel A of Fig 5. Blood transfusions are unlikely to be given to patients who die very rapidly after admission, because of the time required to cross-match blood and then set up a transfusion. This selection bias can be reduced by stratifying by time to death. This information was recorded in all patients. We assume that patients surviving longer than 4 hours postadmission (randomisation) have probabilities of receiving transfusions conditionally independent of their time to death, conditional on study site and baseline covariates. The value of 4 hours is based on previous detailed reports of time to transfusion [41].

Blood transfusion status was only recorded reliably in the AQUAMAT, AAV, and AQ studies (*n* = 6,307, with no missing values). An analysis stratified by time to death limited to these three studies did not show a statistically significant protective effect for transfusion, with an adjusted odds ratio of 0.90 (0.72–1.63). This can be compared with the ‘naive’ analysis not accounting for the competing risk of death, which gives a substantially lower adjusted odds ratio of 0.61 (0.49–0.78). The estimate of the direct effect of haematocrit was approximately the same as for the non-mediated analysis but was not statistically significant, with an adjusted odds ratio of 0.91 (0.79–1.04); see S2 Fig.

We explored the relationship between transfusion and death further in a ‘moderate anaemia’ range, defined ad hoc as 15%–25% haematocrit, using the data from the AQUAMAT study. The causal effect of transfusion was estimated using IPW with propensity scores (for a recent review, see [33]). In this important subgroup of patients who are above the standard thresholds for transfusion (*n* = 2,214), there was no evidence for a beneficial effect of transfusion, with an odds ratio for death in the transfused group of 0.99 (95% CI 0.97–1.02). S3 Fig shows the standardised differences between the transfused and not-transfused groups before and after IPW for all the main recorded clinical variables.

The assumption of no unmeasured confounders for the relationship between anaemia and death may be incorrect. A simplified DAG in panel B of Fig 5 shows two possible confounding effects. First, anaemia at presentation may be a proxy marker for a longer time course of illness [42]. In this scenario, death is a less likely outcome (other more rapidly lethal processes having been avoided) compared with a patient with fulminant complications. Second, anaemia may be a proxy marker for unobserved genetic polymorphisms (such as G6PD deficiency or haemoglobinopathies, which both cause anaemia directly and protect against death in patients with severe malaria [37]). The e-value of the observed adjusted odds ratio of anaemia on mortality is 1.53 (lower bound: 1.25) [43]. This implies that the unmeasured confounder would need to increase the risk of anaemia (as measured by a decrease of 10 absolute percentage points in haematocrit) and of mortality by approximately 1.53-fold for unmeasured confounding to explain the observed protective relationship of haematocrit on mortality. It is difficult to put this number into context given the lack of cohort studies sufficiently powered to identify individual predictors of mortality at the community healthcare level. For example, haemoglobin S has an extremely strong protective effect against mortality from severe malaria (and in haemoglobin SS homozygotes is associated with reduced steady-state haemoglobin concentrations), but this has resulted in very few heterozygous and homozygous patients having been enrolled in severe malaria studies.

## Discussion

In the absence of randomisation, causal inference is difficult and relies on nonstatistical assumptions supported by expert knowledge. Formalising these assumptions using causal diagrams (DAGs), which are readily understood and therefore readily challenged, improves transparency and facilitates investigation. Using a structural causal models framework [13], direct or total causal effects of the main features of severe malaria (anaemia, coma, acidosis, seizures, pulmonary oedema, AKI) on death in patients with severe falciparum malaria were examined, based on a pooled analysis of patient data from 9,040 prospectively studied children and adults in Southeast Asia and sub-Saharan Africa. This allowed for causal interpretation of factors that have been reported previously to be associated with increased risk, but it also suggested that moderate anaemia might confer some survival advantage in patients with severe falciparum malaria.

If it is indeed true that ‘moderate anaemia’ (e.g., haematocrits below the median values: 23% in the pooled dataset, 20% in African patients, and 30% in Asian patients) reduces the mortality of patients with severe falciparum malaria, the mechanisms of the protective effect are unclear. In severe malaria, macrocirculatory changes are characterised by an increase in cardiac output and a moderate decrease in peripheral vascular resistance so that severe hypotension is rare [44]. However, microcirculatory flow is severely compromised, with an increase in microcirculatory resistance caused by sequestered parasitised red cells, generally increased erythrocyte rigidity, and clumping of unparasitised and parasitised erythrocytes (rosetting and autoagglutination, respectively) [45,46]. Furthermore, there is evidence that compensatory endothelial vasodilation is disturbed, related to nitric oxide depletion [47]. Anaemia reduces cytoadherence [48]—the pathological process leading to sequestration—and potentially decreases rosette formation (intererythrocytic adhesion). Anaemia also reduces blood viscosity, in particular under the low-shear conditions at the venular side of the circulation, where sequestration begins [49]. Thus, moderate anaemia could improve blood flow in the partially obstructed microvasculature. The optimal haematocrit, a trade-off between oxygen carriage and blood viscosity, might be lower in patients with severe falciparum malaria, and this could, in part, contribute to the observed unadjusted parabolic relationship between mortality and haematocrit (reference [42] and Fig 2, top middle). Planned studies that measure the capillary haematocrit directly and also determine oxygen delivery in relation to the level of anaemia in severe malaria will be important to support or refute these hypotheses.

If moderate anaemia does confer protection against death, it would have significant implications for patient management and, particularly, for blood transfusion, suggesting a more conservative transfusion strategy may be warranted. It should be noted that, whereas transfused red cells may have less immediate oxygen-carrying capacity (depending on their oxygen dissociation curves), they may be more deformable than the relatively rigid erythrocytes that occur in severe malaria. The transfused plasma may also have antimalarial activity in endemic areas. Thus, the effects of blood transfusion on microvascular perfusion may not be predicted accurately by the relationship between haematocrit and oxygen delivery in severe falciparum malaria. The mediation analysis in this study lacked power to determine whether the protective effect of lower haematocrits was partially mediated by blood transfusion or not. However, in the reduced pooled data with reliably collected transfusion data and stratified by time to death, there was not strong evidence that blood transfusion was associated with a reduced risk of mortality. This stratification is necessary, as patients who receive transfusions are a selected group who by definition have survived long enough to receive the blood. Moribund patients may be less likely to be transfused. Resolution of this important management question requires prospective studies assessing blood transfusion thresholds.

Our data do not contain information on host genetic markers, but the hypothesis of a protective effect of anaemia in severe malaria could be tested on recent large pooled datasets that have collected these data [50]. Treatment-seeking behaviour is a very important determinant of outcome in a rapidly fatal infection, but adjustment for this effect is extremely difficult, as all detailed severe-malaria studies are hospital or clinic based. Further confirmation or refutation of this anaemia hypothesis must be derived from different data sources. For example, an ongoing trial randomising both transfusion and transfusion doses will provide high-quality data addressing this exact question [51].

This study estimated causal effects from observational data (observational with respect to the exposures of interest). The causal interpretation of the relationship between patient covariates and mortality is strongly dependent on the structure of the underlying causal model. The main limitations can be summarised as follows. Unmeasured confounding is plausible, leading to indirect relationships (e.g., moderate anaemia could in fact be a proxy for another true protective variable such as ‘immunity’ or protective genetic haplotypes). A key consideration in the DAG is the role of sepsis in the causal pathway of severe malaria. In the large majority of the pooled data analysed, concomitant sepsis was not assessed by blood culture (which itself is an insensitive measure). This could result in contamination of the data. Sepsis can occur independently of the malarial infection in a patient with incidental asymptomatic parasitaemia, or it can be a result of parasite sequestration (particularly in the gut). This is mainly an issue in the higher-transmission settings in sub-Saharan Africa [52]. If sepsis (caused by the malarial infection) is included in the DAG in Fig 3, then none of the causal effects are identifiable. However, in sub-Saharan African settings, the effect is likely to be small, given that a sensitivity analysis including only data from Asia (40% of the available data) gave very similar results to the overall dataset. Another limitation in the analysis of this dataset is the nonspecific diagnosis of pulmonary oedema in the large AQUAMAT study. Without chest radiographs, pulmonary oedema is difficult to diagnose correctly in children; however, pulmonary oedema is more a problem of adults (particularly pregnant women) than of children. Whereas the specificity of the diagnosis of coma, seizures, shock, and laboratory measures indicating AKI and hypoglycaemia is very high, the specificity of the clinical diagnosis of pulmonary oedema from respiratory distress and abnormal chest auscultation is lower.

Further clinical investigation is necessary to determine whether there is truly any benefit from moderate anaemia in patients with severe malaria and, if so, to characterise the relationship and its time course, as current therapeutic strategies assume only that it is harmful. It should be emphasised that this observation refers only to patients who present with severe malaria and not to patients with uncomplicated malaria. However, it is possible that the apparent life-saving benefit provided by moderate anaemia has occurred earlier in the course of the illness (i.e., before presentation, preventing the development of vital organ dysfunction). A better understanding of the pathological process would inform indications for blood transfusion and hopefully avoid any detrimental effects of overtransfusion in patients with severe falciparum malaria [51]. Clearly, this is of critical importance, as severe anaemia is the major manifestation of severe malaria in high-transmission settings and the main indication for blood transfusion in children in many malaria-endemic areas.

In conclusion, using a causal inference approach to explore the relationships between clinical features and mortality in patients with severe falciparum malaria, it was found that although six well-established measures of vital organ dysfunction were causally associated with an increased risk of death, the direction of the causal effect of anaemia goes against accepted understanding. Moderate anaemia had a relatively small direct protective effect on outcome. Further clinical investigation is required. However, this observation, derived from a very large prospectively studied population across Asia and Africa, does suggest that the current ‘severe anaemia’ criterion for severe malaria, which is a 5 g/dL threshold for children and 7 g/dL for adults, should be reviewed. The application of causal inference-based methodology may be useful in future studies to investigate the direct effects of major complications of severe malaria on mortality. This could help increase the clinical definition of severe malaria, improve the understanding of its pathophysiology, and provide a sound basis for developing and evaluating adjunctive interventions.

## Supporting information

S1 Fig Comparison between Asian and African data. Green diamonds (lines): point estimates (95% CI) using data from only Asian patients. Blue diamonds (lines): point estimates (95% CI) using data from only African patients. Black diamonds (lines): point estimates (95% CI) using the whole pooled database, as shown in Fig 4.
(TIF)

S2 Fig Mediation analysis looking at the effect of transfusion on survival. The forest plot shows the estimated effects on survival of main patient characteristics and treatment drug used, stratified by time to death and adjusted for transfusion.
(TIF)

S3 Fig Standardised differences for the major clinical variables between the transfused and not-transfused children in the AQUAMAT study with baseline haematocrits between 15% and 25%. The black circles show the standardised differences for the original population, and the red triangles show the standardised differences for the IPW population. Standardised differences greater than 0 indicate higher values of that covariate in the transfused group (e.g., base deficit), whereas standardised differences less than 0 indicate higher values in the not-transfused group (e.g., coma). The vertical dashed lines show ±10% standardised difference. AQUAMAT, Africa multinational quinine versus artesunate in severe-malaria trial; IPW, inverse probability weighting.
(TIF)

S1 Table Inclusion criteria for the studies used in the pooled data analysis. HCT, haematocrit; SB, serum bilirubin; SBP, systolic blood pressure; SC, serum creatinine; UO, urine output.
(XLSX)

## Abbreviations

AAV: artemether versus artesunate trial in Vietnam
AKI: acute kidney injury
AQ: artemether versus quinine trial in Vietnam
AQUAMAT: Africa multinational quinine versus artesunate in severe-malaria trial
DAG: directed acyclic graph
G6PD: glucose-6-phosphate dehydrogenase
IPW: inverse probability weighting
QC: quinine versus chloroquine trial in The Gambia
SEAQUAMAT: South East Asian multinational quinine versus artesunate malaria trial
