# Supplementary figures and images for: Investigating causal pathways in severe falciparum malaria: A pooled retrospective analysis of clinical studies
Source: PLoS Med. 2019 Aug 23;16(8):e1002858. doi: 10.1371/journal.pmed.1002858 (PMC6707545; doi:10.1371/journal.pmed.1002858)

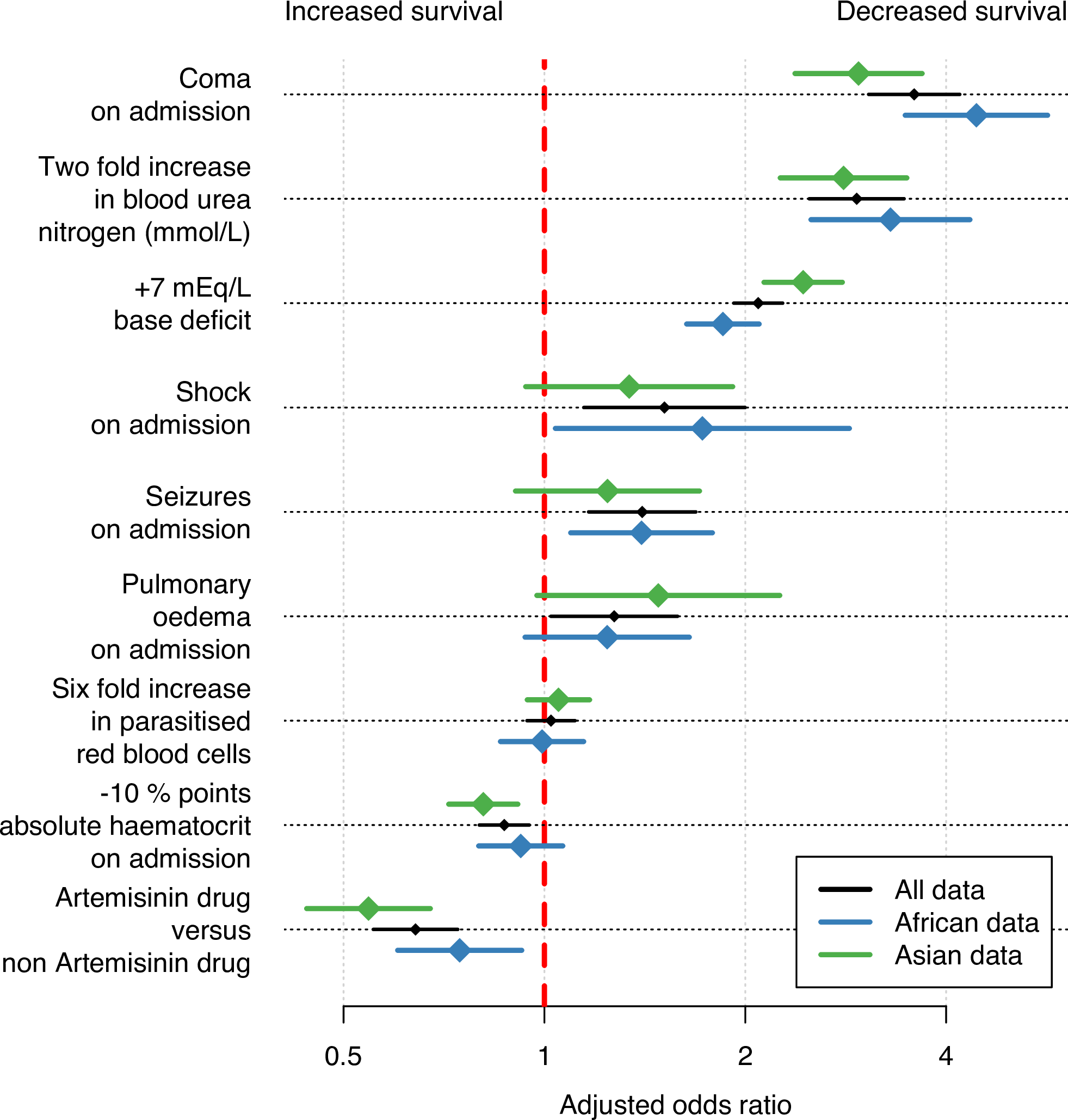

Supplement: S1 Fig — Green diamonds (lines): point estimates (95% CI) using data from only Asian patients. Blue diamonds (lines): point estimates (95% CI) using data from only African patients. Black diamonds (lines): point estimates (95% CI) using the whole pooled database, as shown in Fig 4. (TIF) [file pmed.1002858.s001.tif]

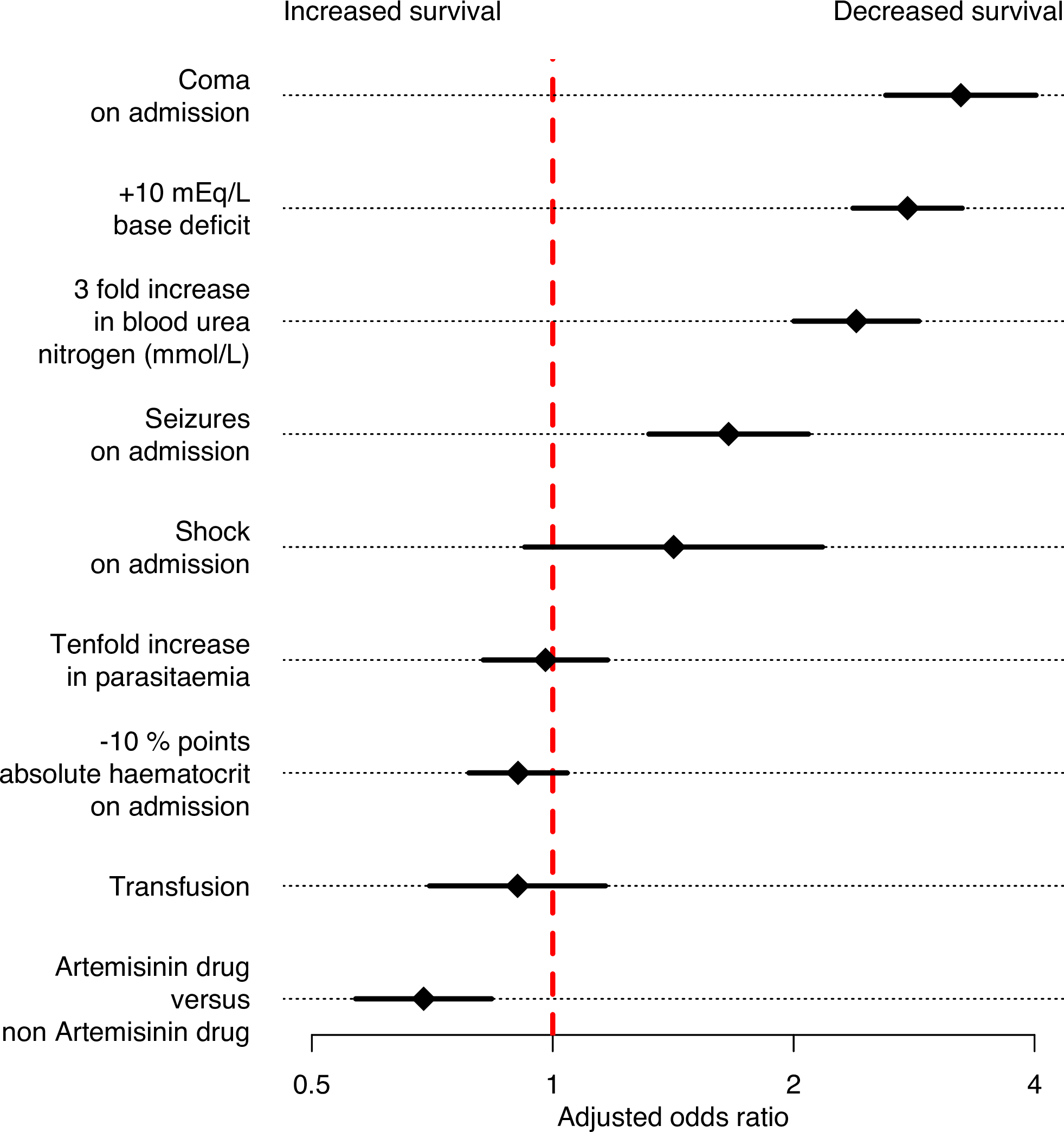

Supplement: S2 Fig — The forest plot shows the estimated effects on survival of main patient characteristics and treatment drug used, stratified by time to death and adjusted for transfusion. (TIF) [file pmed.1002858.s002.tif]

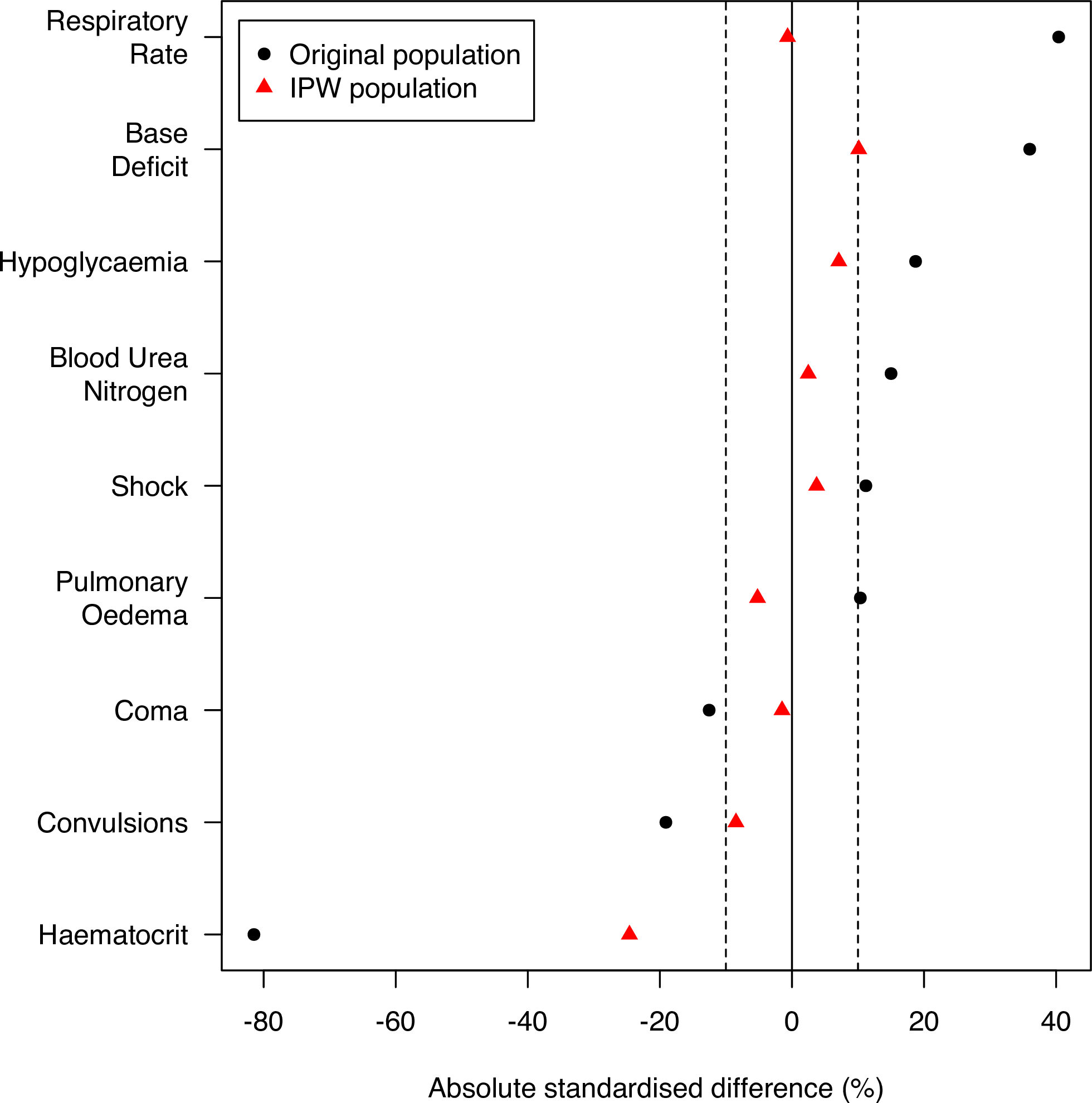

Supplement: S3 Fig — The black circles show the standardised differences for the original population, and the red triangles show the standardised differences for the IPW population. Standardised differences greater than 0 indicate higher values of that covariate in the transfused group (e.g., base deficit), whereas standardised differences less than 0 indicate higher values in the not-transfused group (e.g., coma). The vertical dashed lines show ±10% standardised difference. AQUAMAT, Africa multinational quinine versus artesunate in severe-malaria trial; IPW, inverse probability weighting. (TIF) [file pmed.1002858.s003.tif]
